# Integration of Mobile Health Into Sickle Cell Disease Care to Increase Hydroxyurea Utilization: Protocol for an Efficacy and Implementation Study

**DOI:** 10.2196/16319

**Published:** 2020-07-14

**Authors:** Jane S Hankins, Nirmish Shah, Lisa DiMartino, Donald Brambilla, Maria E Fernandez, Robert W Gibson, Victor R Gordeuk, Richard Lottenberg, Abdullah Kutlar, Cathy Melvin, Jena Simon, Ted Wun, Marsha Treadwell, Cecelia Calhoun, Ana Baumann, Michael B Potter, Lisa Klesges, Hayden Bosworth, Sickle Cell Disease Implementation Consortium

**Affiliations:** 1 St. Jude Childrens Research Hospital Memphis, TN United States; 2 Department of Medicine Duke University Durham, NC United States; 3 Research Triangle Institute Research Triangle Park, NC United States; 4 University of Texas Health Science Center at Houston School of Public Health Houston, TX United States; 5 Center for Blood Disorders Medical College of Georgia Augusta University Augusta, GA United States; 6 Division of Hematology and Oncology Department of Medicine University of Illinois at Chicago Chicago, IL United States; 7 Division of Hematology/Oncology Department of Medicine University of Florida Gainsville, FL United States; 8 Department of Public Health Sciences College of Medicine Medical University of South Carolina Charleston, SC United States; 9 Ichan School of Medicine at Mount Sinai New York, NY United States; 10 Division of Hematology Oncology UC Davis School of Medicine Davis, CA United States; 11 University of California San Francisco Benioff Children Hospital Oakland Oakland, CA United States; 12 Division of Hematology Oncology Department of Pediatrics Washington University St. Louis, MO United States; 13 University of California San Francisco School of Medicine San Francisco, CA United States; 14 Bethesda, MA United States

**Keywords:** sickle cell anemia, digital medicine, adherence, hydroxycarbamide, RE-AIM, implementation science, health innovation, mobile phone

## Abstract

**Background:**

Hydroxyurea prevents disease complications among patients with sickle cell disease (SCD). Although its efficacy has been endorsed by the National Health Lung and Blood Institute evidence-based guidelines, its adoption is low, both by patients with SCD and providers. Mobile health (mHealth) apps provide benefits in improving medication adherence and self-efficacy among patients with chronic diseases and have facilitated prescription among medical providers. However, mHealth has not been systematically tested as a tool to increase hydroxyurea adherence nor has the combination of mHealth been assessed at both patient and provider levels to increase hydroxyurea utilization.

**Objective:**

This study aims to increase hydroxyurea utilization through a combined two-level mHealth intervention for both patients with SCD and their providers with the goals of increasing adherence to hydroxyurea among patients and improve hydroxyurea prescribing behavior among providers.

**Methods:**

We will test the efficacy of 2 mHealth interventions to increase both patient and provider utilization and knowledge of hydroxyurea in 8 clinical sites of the NHLBI-funded Sickle Cell Disease Implementation Consortium (SCDIC). The patient mHealth intervention, *InCharge Health,* includes multiple components that address memory, motivation, and knowledge barriers to hydroxyurea use. The provider mHealth intervention, *Hydroxyurea Toolbox* (*HU Toolbox*), addresses the clinical knowledge barriers in prescribing and monitoring hydroxyurea. The primary hypothesis is that among adolescents and adults with SCD, adherence to hydroxyurea, as measured by the proportion of days covered (the ratio of the number of days the patient is covered by the medication to the number of days in the treatment period), will increase by at least 20% after 24 weeks of receiving the *InCharge Health* app, compared with their adherence at baseline. As secondary objectives, we will (1) examine the change in health-related quality of life, acute disease complications, perceived health literacy, and perceived self-efficacy in taking hydroxyurea among patients who use *InCharge Health* and (2) examine potential increases in the awareness of hydroxyurea benefits and risks, appropriate prescribing, and perceived self-efficacy to correctly administer hydroxyurea therapy among SCD providers between baseline and 9 months of using the *HU Toolbox* app. We will measure the reach, adoption, implementation, and maintenance of both the *InCharge Health* and the *HU Toolbox* apps using the reach, effectiveness, adoption, implementation, and maintenance framework and qualitatively evaluate the implementation of both mHealth interventions.

**Results:**

The study is currently enrolling study participants. Recruitment is anticipated to be completed by mid-2021.

**Conclusions:**

If this two-level intervention, that is, the combined use of InCharge Health and HU Toolbox apps, demonstrates efficacy in increasing adherence to hydroxyurea and prescribing behavior in patients with SCD and their providers, respectively, both apps will be offered to other institutions outside the SCDIC through a future large-scale implementation-effectiveness study.

**Trial Registration:**

ClinicalTrials.gov NCT04080167; https://clinicaltrials.gov/ct2/show/NCT04080167

**International Registered Report Identifier (IRRID):**

DERR1-10.2196/16319

## Introduction

### Sickle Cell Disease and Hydroxyurea Therapy

Sickle cell disease (SCD) is a genetic disorder affecting approximately 100,000 Americans [1]. The effects of SCD are devastating, including severe acute and chronic pain, cognitive disability, renal failure, and lung disease. In controlled clinical trials, hydroxyurea reduces SCD complications (acute pain and acute chest syndrome events) and costs [2-5]. In uncontrolled population studies, hydroxyurea reduces hospitalizations and mortality, supporting the effectiveness of hydroxyurea outside of research studies [6-11]. Hydroxyurea is prescribed in a once-daily dosing, and blood counts are monitored every 1 to 3 months and titrated to reach a maximum tolerated dose defined by mild, reversible myelosuppression [12]. Given the evidence of its benefit, in 2014, the National Institutes of Health/National Heart, Lung, and Blood Institute (NHLBI) released guidelines recommending the use of hydroxyurea [13].

### Hydroxyurea Underutilization and Efforts to Improve Its Use

Despite overwhelming evidence of its positive effects, hydroxyurea is vastly underutilized [14,15]. In analyses conducted using Medicaid claims data, fewer than 50% of adults were ever prescribed or initiated hydroxyurea, and only about 30% of those who initiated treatment achieved adequate adherence levels [16-19]. Among children, adherence was higher; however, the number of children who were prescribed hydroxyurea was low [20-22]. Barriers to prescribing hydroxyurea include providers’ reluctance due to lack of knowledge about the drug and appropriate dosing, low patient acceptance due to insufficient knowledge or misconceptions about risks and benefits, and forgetfulness leading to poor adherence [14,15,20,23-28]. Patient forgetfulness related to daily hydroxyurea use is a common barrier [29] and may be exacerbated by the prevalence of cognitive dysfunction in patients with SCD, including working memory deficits and low motor processing speed [30-32]. Additionally, negative perceptions toward hydroxyurea are strongly associated with lower adherence to this medication [33]. Among prescribers, the anticipation of poor patient adherence dissuades medical providers from prescribing hydroxyurea [19,20,34].

Improved adherence to hydroxyurea achieves higher fetal hemoglobin (HbF) levels (thereby decreasing polymerization of the sickle hemoglobin), fewer hospital admissions, a higher health-related quality of life, and reductions in health care costs, resulting in major improvements in overall clinical outcomes [35,36].

In an effort to address the underutilization of evidence-based recommendations, including that of hydroxyurea, the NHLBI established the Center for Translation Research and Implementation Science in 2014 [37], and in 2016, the NHLBI funded the SCD Implementation Consortium (SCDIC) [38]. The goal of the SCDIC is to support multilevel and multicomponent interventions to address the quality gap in the delivery of evidence-based treatments for patients with SCD between the ages of 15 and 45 years (when the gap in care delivery is the greatest) employing implementation science strategies. The Integration of mHealth into SCD Care to Increase Hydroxyurea Utilization study is one of the planned multicenter studies within the SCDIC that utilizes the implementation science framework and evaluation strategies to increase the adaptation and dissemination of evidence-based treatments among individuals with SCD.

### Mobile Health Technology and Its Potential for Sickle Cell Disease Care and Hydroxyurea Utilization

The existing body of research provides support for mHealth interventions to improve treatment adherence across a variety of chronic conditions, including SCD [39-42]. Among patients with SCD, approximately 85% to 97% of patients own smartphones [29,43], and some use this technology to monitor pain [44,45].

Preliminary studies suggest that mHealth interventions can specifically be used to improve hydroxyurea adherence. In children with SCD, the use of text message reminders combined with direct-observed therapy (via video recording) and financial incentives for 6 months significantly increased hydroxyurea adherence and hematologic markers [46,47]. In a study of 81 adolescents with SCD who received text messaging to improve hydroxyurea adherence, significant increases in relevant hematological indices (HbF, mean corpuscular volume [MCV], hemoglobin [Hb]) and significant reduction of hemolysis markers (absolute reticulocyte count [ARC], bilirubin, and lactate dehydrogenase [LDH]) were observed [43]. Collectively, these findings suggest that hydroxyurea use can be improved with the use of mHealth via improved adherence. A systematic review of mHealth apps for SCD has confirmed these findings but observed that the sample size of most studies was not large, and the studies were mostly observational or retrospective [42].

mHealth is also increasingly used to aid physicians in their medical decision making [48] and to facilitate consultations with other providers and experts in their areas of expertise [49,50], highlighting the broad applicability of mHealth, not only for patients but also for medical providers. In a survey of health care providers, more than 70% of physicians had smartphones, and 77% of nurses and doctors used medical apps [51]. The ease of access and increasing familiarity with apps has led to a growing focus on developing disease-specific medical apps for health care providers.

### Logic Model of Change to Increase Hydroxyurea Utilization

**Figure 1:**
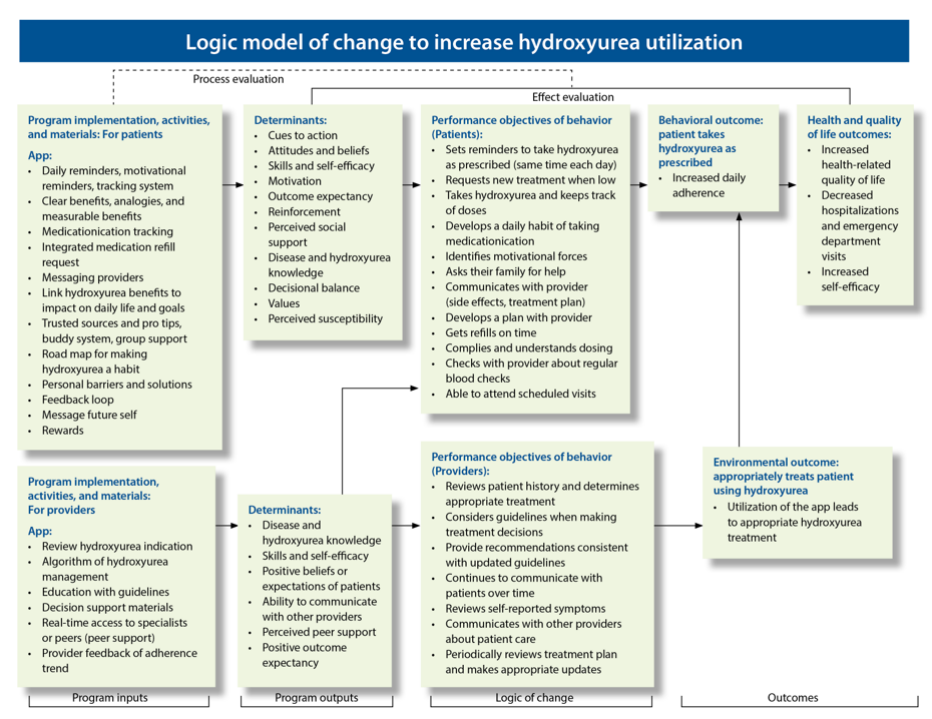
Logic model of change to increase hydroxyurea utilization. This logic model maps all barriers identified by literature review and needs assessment analysis, with a focus on the determinants of the behaviors to hydroxyurea use. The intervention addresses the determinants of hydroxyurea use at both the patient and provider levels. If this two-level intervention is successful, hydroxyurea utilization will increase, as reflected by increased hydroxyurea adherence, resulting in improved health-related quality of life and reduction in acute health care utilization.

We conceptualized a logic model that guided the development of mHealth apps to foster hydroxyurea utilization among patients and improve provider prescribing behaviors. This logic model used intervention mapping methods to develop and adapt behavioral models for testing mHealth as an intervention to increase hydroxyurea use. Intervention mapping is a systematic framework for developing, implementing, and adapting theory- and evidence-based interventions [52]. Using the knowledge of barriers to the uptake and adherence to hydroxyurea therapy, we mapped the determinants of hydroxyurea utilization (Figure 1). These determinants are hypothesized to drive the behaviors involved in patients’ and providers’ use of hydroxyurea and correspond to the barriers of hydroxyurea use that were identified through literature review and the results of a needs assessment within the study participating sites. Importantly, the patient and provider interventions were developed and aimed at the determinants that could affect the behavior involved in taking and prescribing hydroxyurea; the ultimate goal (the behavioral outcome) is to foster greater patient adherence to hydroxyurea (Figure 1).

### Hydroxyurea Adherence Behaviors for Patients

To guide the development of the mHealth app for patients, we used the health belief model (HBM) as the framework [53] for behavioral change. The HBM explains health behaviors and focuses on the attitudes and beliefs of individuals. The health-related action driving the increased use of hydroxyurea includes 5 constructs: perceived susceptibility, perceived severity, perceived benefits, perceived barriers, and self-efficacy. Notably, these 5 constructs represent modifiable factors that, together, can influence the increased use of hydroxyurea. The patient intervention focuses on these 5 constructs as the specific mechanisms to address the change in behavior (ie, medication adherence).

### Behavioral Model for Mobile Health Utilization Among Medical Providers

The acceptance of new technology by users, including new mHealth innovations, determines its successful adaptation and, therefore, its intended effects. The perceptions of health care professionals regarding their ability to use mobile health care systems to accomplish a health care task is an important determinant that should also be considered when new technology is implemented. The technology acceptance model [54,55] is a conceptual model that explains the intent to use new information technology (eg, mHealth) or information science among users, including medical providers. Perceived usefulness, perceived ease of use, compatibility, and mobile health care systems self-efficacy are the most important determinants of the behavior intent of providers [56]. We considered all of these drivers and assessed them in the context of the hydroxyurea prescriber to develop the provider *HU Toolbox* as follows:

- *Perceived usefulness*: SCD providers require concise information to support clinical decision making while prescribing hydroxyurea.
- *Compatibility*: The previous experience SCD providers have in using mobile technology was considered.
- *Perceived ease of use*: The perception of SCD providers that mobile technology can be integrated with their electronic health system and their daily clinical routine.
- *Mobile health care systems self-efficacy*: SCD providers’ perception that mHealth could help with the task of prescribing hydroxyurea.

#### Description of the Mobile Health Interventions

##### InCharge Health Mobile App

*InCharge Health* was developed using a user-centered design approach, in which the patients’ input in its development was obtained through an iterative process that started with a design-thinking session, followed by surveys and interviews that investigated barriers and facilitators of hydroxyurea use and preferences for its use [57]. A prototype was developed and further refined using information derived from focus groups with patients with SCD.

**Figure 2:**
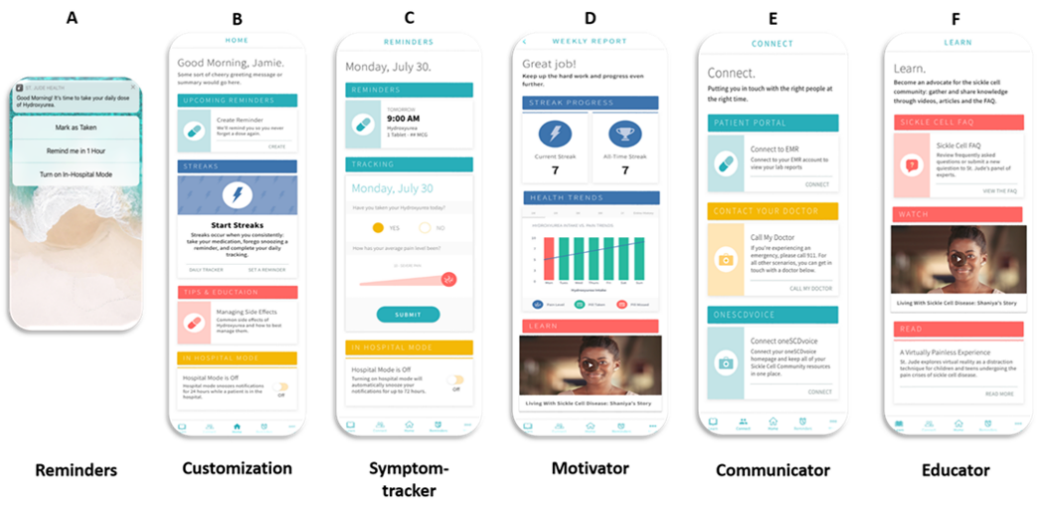
Features of the InCharge Health app for patients. (A) Push notifications will come daily and will prompt the patient participant to mark if the dose was taken, not taken, or be reminded later. (B) Customization of the push notification messages, time of the day, and choice of the accountability partner. (C) A daily pain and mood tracker is available and captures pain level and mood changes. (D) Graphing of pain level versus pain is available for the past 7 days. (E) A link to the patient portal accesses the patient’s electronic medical chart, clinic numbers, and patient-led discussion forums. (F) A large resource bank is available with links to vetted educational websites, educational material, and educational videos and is included.

The *InCharge Health* mobile app includes several features to increase patient engagement, including the following: (1) daily customizable text message reminders that can be sent if the patient is hospitalized, (2) a daily recording of hydroxyurea adherence and pain score, (3) a 7-day streak that tracks daily adherence and graphing of adherence against pain symptoms, (4) a communication feature that allows the patient to connect to the health care provider and other patients, and (5) an education bank that provides information about SCD and hydroxyurea risks and benefits in layman’s terms (Figure 2). Additionally, *InCharge Health* has an accountability partner feature that specifies a person (eg, friend, family member) who will receive notifications if the user has not documented the use of hydroxyurea for >4 hours (from the time of receipt of the daily reminder) and is encouraged to remind the patient to take their medication. The *InCharge Health* app does not collect any protected health information and functions in places with Wi-Fi accessibility or using the phone’s data plan.

##### Hydroxyurea Toolbox Mobile App

**Figure 3:**
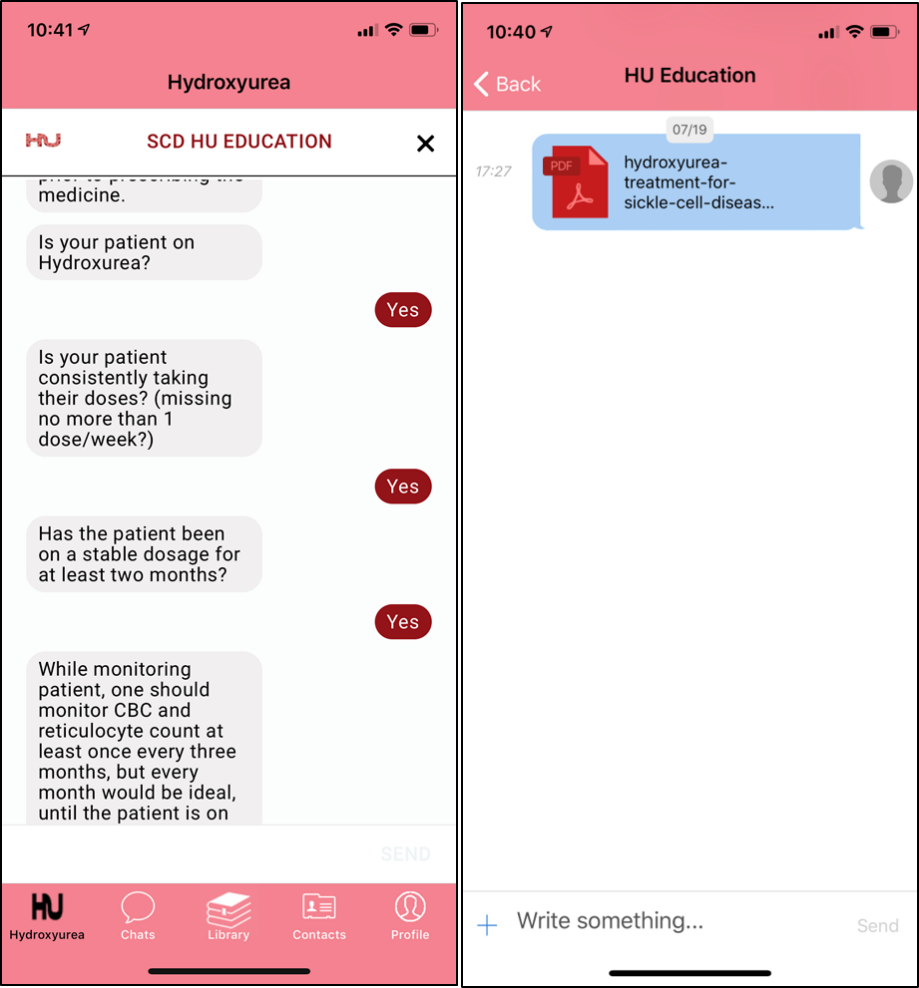
Features of the HU Toolbox mobile app: The HU Toolbox provides tools for medical providers prescribing hydroxyurea. The app provides a chatbot for users to ask questions about how to dose and monitor hydroxyurea effects and side effects (left panel). In addition, there is educational material available in a library (right panel) as well as the ability to chat directly with a local SCD specialist. HU Toolbox: Hydroxyurea Toolbox; SCD: sickle cell disease.

The *HU Toolbox* is a decision-support tool developed with input from pediatricians, internists, and hematologists from several academic centers in North Carolina and members of the Community Care of North Carolina medical home system. The *HU Toolbox* app contains NHLBI guidelines adapted for pediatric and adult providers (guidelines and recommendations stratified by age). In addition to being an information source, the app contains artificial intelligence algorithms guiding the clinician on how to prescribe hydroxyurea and monitor its effects through a chatbot feature, which simulates a human conversation using text messaging. The *HU Toolbox* chatbot feature guides clinicians on how to recognize hydroxyurea side effects and how to manage them (Figure 3). Finally, a built-in SCD specialist feature is available, allowing providers to reach and consult SCD experts in their region, who respond to enquiries within 24 hours.

### Specific Aims and Objectives

#### Aim 1: Improve Patient Adherence to Hydroxyurea

We will compare adherence to hydroxyurea at baseline with adherence after 6 months of *InCharge Health* among adolescents and adults with SCD. Our primary hypothesis is that, among patients with SCD, hydroxyurea therapy adherence will increase by 20% at 24 weeks after receiving the *InCharge Health* intervention, compared with their hydroxyurea adherence measured at baseline. Adherence will be measured by the proportion of days covered (PDC; the ratio of the number of days the patient is covered by the medication to the number of days in the treatment period) [58]. The rationale for choosing the primary end point is because a 20% increase in PDC is a clinically meaningful change and represents an increment of approximately 1.4 additional days of hydroxyurea use in a week’s period. Our conservative estimated increase of 20% refill is based on previous studies that used text messages to increase hydroxyurea adherence and observed adherence increases as high as 60% [59]. The timing of the primary end point (at 24 weeks) is such that it will allow sufficient time to observe clinical and laboratory changes from increased hydroxyurea adherence, as it may take an average of 4 to 6 months to observe full hydroxyurea effects.

#### Aim 1a: To Assess Patient Engagement and Behaviors Related to Use of InCharge Health

We will evaluate the consistent use of the app, patient satisfaction, and continued use of the app beyond the study period.

#### Aim 1b: To Examine the Improvement in Clinical and Patient Outcomes Related to the Use of InCharge Health

We will investigate changes in the proportion of patients with PDC >80%, hematologic indices, acute health care utilization, health-related quality of life, and perceived self-efficacy for medication use between baseline and 24 weeks after receiving the InCharge Health intervention.

#### Aim 2: Improve Provider Hydroxyurea Prescribing Behaviors

Among providers using the HU Toolbox app, we will examine the changes in knowledge of hydroxyurea benefits and risks as well as perceived self-efficacy to correctly prescribe hydroxyurea therapy between baseline and after 9 months of using the HU Toolbox intervention.

#### Aim 2a: To Examine Clinical Characteristics and Provider Engagement and Behaviors Related to the Use of the HU Toolbox

We will evaluate the frequency with which providers use the app and provider satisfaction and continued use of the app beyond the study period.

#### Aim 2b: To Assess Combined Effects of the Patient and Provider Mobile Health Interventions on Hydroxyurea Adherence and Acute Health Care Utilization.

We will examine if the changes in hydroxyurea adherence, emergency department visits, and hospitalizations are enhanced by the use of InCharge Health and HU Toolbox concomitantly.

#### Aim 3

We will qualitatively evaluate the barriers and facilitators of the implementation of mHealth interventions. We will examine the strategies used to support the implementation of mHealth interventions and evaluate the facilitators and barriers to implementation from multiple stakeholder perspectives: patients, providers, and administrators.

## Methods

The study protocol is reported in accordance with the Standard Protocol Items for Clinical Trials (SPIRIT), where applicable (SPIRIT checklist; Multimedia Appendix 1) [60].

### Evaluation Framework

Key considerations to begin implementing mHealth for hydroxyurea utilization include recruitment in diverse care settings and estimating the reach, effectiveness, adoption, implementation, and maintenance of the apps, which are the 5 components of the RE-AIM framework [61]. RE-AIM is a useful framework to evaluate the utility of mHealth to foster hydroxyurea utilization and to broaden the future applicability and dissemination of the apps [62,63]. RE-AIM will be used in this study to measure the overall impact and robustness of apps to achieve improved patient adherence to hydroxyurea and better prescribing practices of this drug.

### Study Design

**Figure 4:**
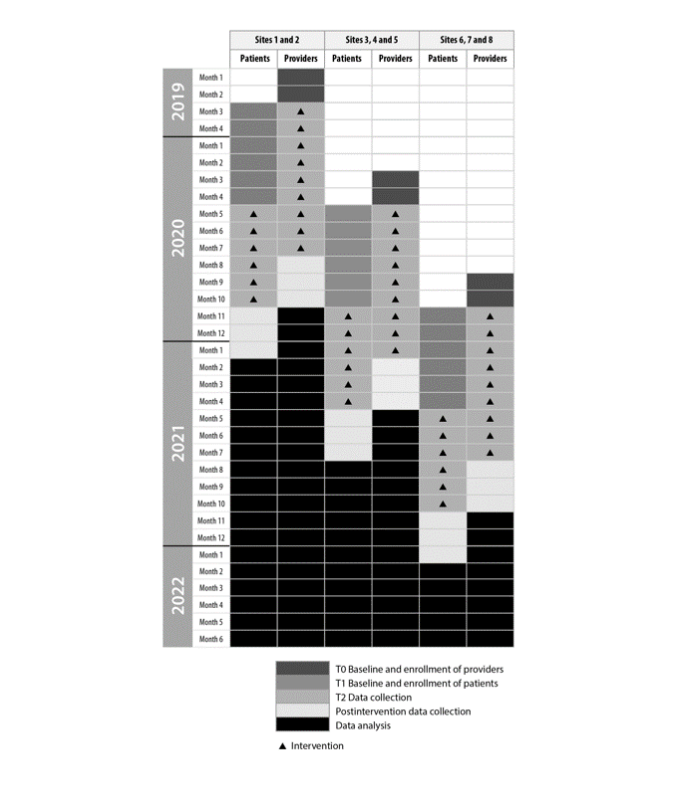
Study time periods: 2 or 3 groups of sites will enter the study at each of the 3 time periods, and 4 study phases will take place during each study period. T0: introduction of HU Toolbox to providers, provider enrollment, and baseline data collection. T1: introduction of InCharge Health to patients, patient enrollment, and baseline data collection. T2: All enrolled patients and provider participants were followed as active study participants. Each patient will use the InCharge Health app for 6 months, and each provider will use the HU Toolbox app for 9 months. Postintervention data collection: this phase reflects the sustainability of the interventions. We will continue to provide technical support for both patient and provider applications and measure continued utilization of the applications and adherence to hydroxyurea. Solid triangle denotes InCharge Health and HU Toolbox interventions. HU Toolbox: Hydroxyurea Toolbox.

The study design is a nonrandomized, closed cohort trial where the 2 mHealth apps will be introduced sequentially in 8 participating clinic sites over 3 time periods (Figure 4). A cohort of subjects recruited from within each site will be followed over each time period in which the unit of analysis will be the patient. Within each site, there will be one or more treatment clinics.

Each provider within a participating clinic will receive the *HU Toolbox* intervention for 9 months, while each patient participant will receive the *InCharge Health* app intervention for 6 months. The providers (physicians and advance care practitioners) will begin receiving the provider app 2 months before patients (at the same site) initiate the use of the patient app. There will be a staggered 6 months between groups of sites (Figure 4). The study rollout will allow for a baseline evaluation, followed by preparation and introduction of the provider app (education of providers and remaining staff), followed by implementation of the apps, and evaluation postimplementation (Figure 4). Implementing the interventions at the first 2 sites will allow us to determine any challenges and adapt to ensure increased uptake and implementation for the following sites.

### Study Setting

The study will be carried out at 8 diverse SCDIC participating clinical sites and their subsites. The study settings are variable and include academic and nonacademic sites within urban, suburban, and rural settings. The context for the program is diverse and presents an opportunity to test mHealth in different settings, with not only geographical but also structural differences using the RE-AIM evaluation framework.

### Participants

Potential participants will be approached during a nonemergent clinic visit. The study coordinator will verify that the participant (patient or provider) meets the study eligibility criteria (Textboxes 1 and 2), and will approach them in person, phone, or via electronic media about enrolling in the study. Participants will sign an informed consent before study participation (unless a waiver of consent is granted by the local institutional review board). If the participant is a minor, the legal guardian will sign the consent and assent will be obtained. Participants will be considered enrolled when consent is obtained, and inclusion criteria have been confirmed. All providers within each practice will be approached and invited to participate. All clinics will have each provider register within the app to allow provider-specific data. Each site will maintain a local enrollment log and will also confirm enrollment status in the data management system.

**Inclusion and exclusion criteria for patient participants.**

Inclusion criteria

- Between 15 and 45 years
- Treated at or affiliated with one of the sickle cell diseases implementation consortium sites
- English speaking
- Confirmed sickle cell disease diagnosis by a hemoglobin fractionation test
- Owns a cellular/mobile smartphone (either Android or iOS)
- Hydroxyurea therapy:
  - Already receiving hydroxyurea therapy (at least one previous prescription for hydroxyurea in the past 3 months) or
  - Initiating hydroxyurea therapy (the first prescription must be written on the same day as study enrollment). Patient participants who initiate hydroxyurea on the same day of study enrollment will not contribute to the total target accrual for the site, but rather will be analyzed as a separate group

Exclusion criteria

- Known current pregnancy
- A red blood cell transfusion in the past 60 days. This is necessary as transfusions will mask laboratory markers and clinical changes from hydroxyurea
- Currently using consistency. another cellphone app or a web-based tool (electronic health tool) to increase hydroxyurea adherence

**Inclusion criteria and the exclusion criterion for provider participants.**

Inclusion criteria

- Physician (including physician in training) or advanced practice provider (nurse practitioner or physician assistant) who cares for at least one patient with sickle cell disease for an anticipated minimum of 12 months from study enrollment
- Access to a cellular/mobile smartphone (either Android or iOS) or access to a computer with internet connectivity (*HU Toolbox* app can be accessed via the internet on any device)
- Exclusion criterion
- Currently using another phone app or a web-based tool to increase hydroxyurea adherence for patients with SCD in his/her practice

#### Implementation Strategies

To promote the uptake of both the patient and provider mHealth apps into practice, we will employ multiple implementation strategies. Sites will be provided with a list of discrete strategies (eg, education strategies such as conducting ongoing training and regular check-ins with patients and providers regarding app functionality). Each clinical site will be able to select a strategy or strategies that best fit their context. All sites will be required to provide training on the apps for both patients and providers using a standard training protocol, which is detailed in the study standard operating procedure document. Research staff will guide the participants on the installation of *InCharge Health* and *HU toolbox* on the participants’ mobile devices (or computer in the case of providers). Using a developed script, the research team will provide instructions about app settings, explain how to use the app, and answer any questions. Before participants leave the clinic, patients will be asked to demonstrate their knowledge and ability to use the app. Centralized technical assistance (by the app developers) will be provided for both apps to ensure a high level of fidelity in their implementation. Specifically, patients and providers will be given a number to call and an email address if they have questions regarding the app or the study in general. Data related to technical problems related to the apps will be tracked to evaluate their functionality.

#### Study Outcome Measures

#### Primary Outcome

Efficacy will be determined by assessing the impact of *InCharge Health* on hydroxyurea by measuring the change in PDC from baseline (before intervention) to week 24 (primary outcome). For the PDC, the pharmacy that fills the most prescription claims within the target therapeutic category for a specific patient within the calendar range will be assigned responsibility for the patient. The pharmacy name and number where hydroxyurea is filled will be collected by the research coordinators at each study visit, and refill information will be requested from these pharmacies. All prescription drug refills, from all dispensing pharmacies, will be ascertained.

#### Secondary Outcomes

These include daily recorded adherence on the app; proportion of patients with PDC ≥80%; laboratory markers of hydroxyurea response (HbF, Hb concentration, MCV, ARC, absolute neutrophil count, indirect bilirubin, LDH); health care utilization (hospitalizations and emergency department visits); health-related quality of life; perceived health literacy, patient medication self-efficacy, and implementation outcomes.

#### Data Collection

After enrolling, patient participants will return at 12 and 24 weeks for study visits, where study-related procedures will be conducted (Table 1). Provider participants will complete an assessment of self-confidence in prescribing hydroxyurea at baseline and at the study exit (Table 2). Three months after study completion, *InCharge Health* and *HU Toolbox* usage will be assessed via analysis of app statistics. Additionally, at study completion, we will solicit feedback from patients and providers regarding the clinical usefulness of the apps and their usability and impact. (Tables 1 and 2). Clinical and implementation measures will be assessed using the RE-AIM framework, both for *InCharge Health* (Table 3) and *HU Toolbox* (Table 4).

We will conduct 15 semistructured key-informant interviews (30-60 min) with multiple key stakeholders toward the end of app implementation at each of the study sites. We will purposively sample and interview patients and providers (physicians and advance care practitioners) from each site according to mHealth app use (low uptake vs high uptake). The RE-AIM framework was used to develop the interview guides and systematically assess barriers and facilitators. For example, patients will be asked how using *InCharge Health* impacts the way they take hydroxyurea, whereas providers will be asked how using the *HU Toolbox* impacts the way they prescribe hydroxyurea. Patients and providers will also be asked about why they chose to participate in the study, training received for the apps, and whether they would continue to use the apps after the study is complete. We also plan interviews with clinic administrators to gain a clinic-level perspective on factors that influenced implementation.

**Table 1:** Schedule of evaluations for patient participants.

Measures and definition				Week 24 (retrospectively collected)		Baseline		Week 12		Week 24 (study exit)		Week 36 (poststudy)
**Socio** **d** **emographic**												
	Age, sex, race, ethnicity, marital status, educational attainment, health insurance type, income, occupation		—^a^		x^b^		—		—		—	
Informed consent				—		x		—		—		—
**Patient adherence to hydroxyurea (Aim 1) and the combined effects of the patient and provider mHealth** ^c^ **interventions (Aim 2b)**												
	**Hydroxyurea adherence**											
		Proportion of daily coverage	x		x		x		x		x	
		App daily adherence statistics and 7-day recall measure using the brief medication questionnaire	—		x				x		—	
**Clinical influence of the InCharge Health app; (Aim 1b) and the combined effects of the patient and provider mHealth interventions (Aim 2b)**												
	**Hydroxyurea effect**											
		Date hydroxyurea initiated	—		x		—		—		—	
		MTD^d^ dose (mg/kg/day) and date reached	—		x		—		—		—	
		Current dose (mg/kg/day and mg/day)	—		x		x		x		x	
		Biomarkers of hydroxyurea effect (HbF^e^%, Hb^f^, MCV^g^, ANC^h^, ARC^i^, indirect bilirubin, LDH^j^)	x		x		x		x		x	
**Health care utilization**												
	Date and discharge diagnosis of ED^k^ visits, acute care/infusion visit hospitalizations		x		x		x		x		x	
**Self-efficacy and health literacy**												
	PROMIS^l^ self-efficacy for medication short form		—		x		—		x		—	
	Perceived health literacy		—		x		—		x		—	
**Health-related quality of life and pain report**												
	ASCQ-Me^m^ pain impact, ASCQ-Me pain episode frequency and severity, PROMIS pain quality		—		x		—		x		—	
**Engagement of patients related to the use of InCharge Health app (Aim 1a)**												
	**Implementation measures**											
		See Tables 3 and 4	—		—		—		x		—	
	**mHealth satisfaction**											
		Perceived usability and acceptability of mHealth intervention (MARS^n^) [64]	—		—		x		x		—	
**Evaluation of facilitators and barriers to implementation of the mHealth app (Aim 3)**												
	**Barriers and facilitators to implementation**											
		Qualitative interviews^o^	—		—		—		x		x	

^a^Denotes not done at this timepoint.

^b^x denotes done at this timepoint.

^c^mHealth: mobile health.

^d^MTD: maximum tolerated dose.

^e^HbF: fetal hemoglobin.

^f^Hb: hemoglobin.

^g^MCV: mean corpuscular volume.

^h^ANC: absolute neutrophil count.

^i^ARC: absolute reticulocyte count.

^j^LDH: lactate dehydrogenase.

^k^ED: emergency department.

^l^PROMIS: Patient-Reported Outcomes Measurement Information System.

^m^ASCQ-Me: adult sickle cell quality of life measurement information system.

^n^MARS: mobile app rating scale.

^o^Conducted at the end of the study at each site.

**Table 2:** Schedule of evaluations for provider participants.

Measure and definition				Baseline		Week 36 (study exit)					Week 48 (poststudy)	
**Sociodemographic**												
		Age, sex, race, ethnicity, type of professional (physician, nurse practitioner, physician assistant), years in practice		x^a^		—^b^					—	
Informed consent				—		—					—	
**Improve provider hydroxyurea awareness, prescribing, and monitoring behaviors (Aim 2)**												
	**Self-efficacy and hydroxyurea knowledge**											
			Perceived confidence in prescribing hydroxyurea to patients with SCD^c^, including correct daily dosing		x		x					—
**Engagement of providers related to the use of the HU Toolbox app (Aim 2a)**												
	**Implementation** **and mHealth** **satisfaction**											
			Perceived usability and acceptability of mHealth intervention (MARS^d^ scale) [64]		—		x					—
	**Hydroxyurea prescribing practices (clinic-level measures)**											
			Total number of patients with SCD		x		x					x
			Number of patients eligible to receive hydroxyurea therapy at provider participant’s site^e^		x		x					x
			Number of hydroxyurea-eligible patients who are prescribed hydroxyurea (all sickle genotypes)^e^		x		x					x
**Evaluation of facilitators and barriers to implementation of the mHealth app (Aim 3)**												
	**Barriers and facilitators to implementation**											
			Qualitative interviews^f^		—				x		—	

^a^x denotes done at this time point.

^b^Denotes not done at this time point.

^c^SCD: sickle cell disease.

^d^MARS: mobile app rating scale.

^e^Hydroxyurea eligibility will follow the 2014 National Health Lung and Blood Institute guidelines as follows: hydroxyurea should be offered to all children with homozygous sickle hemoglobin mutation (HbSS) and compount heterozygous sickle hemoglobin and null beta thalassemia (HbSβ^0^-thalassemia) age ≥9 months and prescribed to all symptomatic adults with HbSS/HbSβ^0^-thalassemia, that is, >3 episodes of severe vaso-occlusion in the preceding 9 months [13].

^f^Conducted at the end of the study at each site.

For additional information on Implementation and mHealth satisfaction, please refer to Tables 3 and 4

**Table 3:** The reach, effectiveness, adoption, implementation, and maintenance evaluation measures of InCharge Health implementation.

Domains and measures		Data sources
Reach	Sociodemographic characteristics of patients at each site Proportion and representativeness of patients screened for the study (numerator) among all patients who receive hydroxyurea treatment (denominator) at each site Proportion and representativeness of patients eligible for the study (numerator) among all patients who receive hydroxyurea treatment (denominator) at each site Proportion and representativeness of patients participating/enrolled in the study (numerator) among all patients who receive hydroxyurea treatment and were eligible (denominator) at each site	Clinic data collection forms Clinic population demographics and treatment data, study database Screening log Qualitative interviews
Effectiveness	Primary outcome >20% improvement in the PDC^a^ for hydroxyurea among those receiving the intervention	Prescription drug claims (PDC for hydroxyurea refills)
	Secondary outcomes Change in Quality of life, self-efficacy, perceived health literacy Change in percentage of patients with ED^b^ visits, hospitalizations since the last study visit Change in biomarkers of hydroxyurea effect (MCV^c^, ANC^d^, ARC^e^, indirect bilirubin, HbF^f^, Hb^g^, LDH^h^)	Patient surveys (ASCQ-Me^i^, PROMIS^j,^ Perceived health literacy, self-efficacy) Medical chart abstraction Qualitative interviews
Adoption	Proportion and description of clinics in each site agreeing to support *InCharge Health* Proportion and description of providers in each clinic agreeing to support *InCharge Health* (ie, proportion enrolled on the study)	Clinic administrative data and data collection forms Qualitative interviews
Implementation	Consistency with which sites are able to implement the app as planned Qualitative assessment of any adaptations or enhancement to recruitment strategies needed to meet enrollment by the clinic, by site Assess adaptation of training needed to improve *InCharge Health* implementation at each site Engagement with the app: percentage, number, and representativeness of patients who used *InCharge Health* during the study period (low, medium-low, medium, or high use; in the entire practice) Proportion, number, and characteristics of patients who complete the study among those who initiate the use of the app but then later discontinue at each site Percentage and characteristics of patients who reported satisfaction with the *InCharge Health* app (MARS^k^ scale) Clinic/provider assessment of perceptions of *InCharge Health* app for further scale-up or sustainability—ease of use, preferred features, and so on	App usage statistics Patient surveys Qualitative interviews
Maintenance/sustainability	Extent to which program leaders express a desire or intent to continue providing the app with patients at the conclusion of the research Percentage of patients who continue to use the app beyond the study period and their representativeness	Pharmacy claims data (PDC for hydroxyurea refills) App use statistics Clinic data collection forms Qualitative interviews

^a^PDC: proportion of days covered.

^b^ED: emergency department.

^c^MCV: mean corpuscular volume.

^d^ANC: absolute neutrophil count.

^e^ARC: absolute reticulocyte count.

^f^HbF: fetal hemoglobin.

^g^Hb: hemoglobin.

^h^LDH: lactate dehydrogenase.

^i^ASCQ-Me: adult sickle cell quality of life measurement information system.

^j^PROMIS: Patient-Reported Outcomes Measurement Information System.

^k^MARS: mobile app rating scale.

**Table 4:** The reach, effectiveness, adoption, implementation, and maintenance evaluation measures of Hydroxyurea Toolbox implementation.

Domains	Measures	Data sources
Adoption—clinic	Proportion and representativeness of clinics that agree to support the *HU*^a^ *Toolbox*	Institutional data to describe clinics (eg, size, case mix, years in service, regional sociodemographics of SCD^b^ patients) Clinic data collection form Qualitative interviews
Adoption—provider	Characteristics of providers at each site (eg, specialty, years in practice, sociodemographics, level of expertise) Proportion and representativeness of eligible providers approached in the study (numerator) among all providers (denominator) Proportion and representativeness of enrolled providers in the study (numerator) among all eligible providers (denominator) at each site	Provider survey Clinic data collection form
Effectiveness	Number and proportion of providers demonstrating improved knowledge and self-efficacy in hydroxyurea administration Percentage of patients who were prescribed hydroxyurea per provider	Provider survey Medical chart abstraction Qualitative interviews
Implementation	Consistency with which sites are able to implement the use of the Toolbox app as planned Engagement with the app: Percentage, number, and representativeness of providers that appropriately used *HU Toolbox* app (low or high use; in the entire practice) Percentage of providers who reported satisfaction with *HU Toolbox* app (MARS^c^ scale) Percentage of patients whose provider used the Toolbox at each site App usage statistics	App usage statistics Provider survey Qualitative interviews
Maintenance/sustainability	Extent to which program leaders express a desire or intent to offer or encourage the use of the Toolbox app by their clinical providers at the conclusion of the research Percentage of providers who continue to use the provider app beyond the study period, and representativeness Percentage of providers who continue to prescribe hydroxyurea to their patients	App usage statistics Clinic data collection form Qualitative interviews

^a^HU: Hydroxyurea*.*

^b^MARS: mobile app rating scale.

^b^SCD: sickle cell disease.

### Sample Size

Sample sizes were calculated for the primary outcome, PDC, using simulations based on the linear mixed model in the analysis plan. First, we modeled the baseline values. Results from Candrilli et al [18] indicated a left-skewed distribution for adherence measured by medication possession ratio. We expect a similarly left-skewed distribution for PDC. The baseline PDC was therefore modeled as PDC=100 × *X*, where *X* follows a beta distribution with parameters 1.0 and 0.6667. This produced a left-skewed distribution with a mean of 60% (SD 30%), closely paralleling a mean of 0.60 (SD 0.32) reported by Candrilli et al [18] for the medication possession ratio. We included site-to-site variation in baseline PDC by adding a site-specific random variate, drawn from a normal distribution with a mean of 0 (SD 0.2) to the 2 parameters. The resulting site-specific means are between 56% and 69%, with a probability of .95. Baseline values were modeled by drawing random samples from these beta distributions.

To model treatment response, including site-to-site variation in response, we added the expected response of 12% plus a site-specific random variable drawn from a normal distribution with a mean of 0 (SD 5.48) to each baseline value for PDC. Residual intrasubject variation at each time point ( in the linear mixed model) was included by adding a separate random variable drawn from a normal distribution with a mean of 0 to each simulated PDC value. Assuming that approximately 25% of 24-week PDC values will be missing, each 24-week observation was randomly deleted with a probability of .25.

The treatment effect is measured against noise, which includes residual intrasubject variation and site-to-site variation in treatment response. Therefore, power was investigated by specifying SDs for the 2 variance components, generating simulated data sets of 8 sites with 46 subjects recruited per site, and fitting the linear mixed model to each data set. Power was estimated as the percentage of simulated data sets producing a statistically significant increase in PDC among 1000 simulated data sets.

Baseline and posttreatment PDC are expected to be moderately to strongly correlated (ie, subjects with lower baseline PDC will tend to have lower posttreatment PDC than those with higher starting values). This expectation places limits on the variance components because the correlation varies inversely with the variance of the measurements. Variance components that result in a correlation of 0.50 also result in approximately 90% power to detect a treatment effect under the conditions specified in the simulations. A correlation of 0.50 is well below expectations, indicating that the study will have considerable power to detect the posited treatment effect under the specified conditions.

Patients who initiate hydroxyurea on the same day of enrollment will not contribute to the primary aim but will be analyzed as a separate subset of participants for the secondary outcomes only, as no baseline PDC will be able to be calculated for them. The total number of physicians and advanced practitioners in all participating sites is approximately 100. All of them will be approached, and the total number of those who agree to participate will be computed.

### Methods of Analysis

#### Aim 1: Improve Patient Adherence to Hydroxyurea

Changes in PDC in response to treatment will be evaluated using a linear mixed model. Predictors will include a random effect for the study site, a fixed binary indicator for treatment, the interaction of the site with treatment, and a random effect for the subject nested within the site. The interaction is included to test for differences in PDC changes among sites. The random subject effect is included to account for the correlation induced by repeated observations of the same subjects. If the interaction is not statistically significant, then it will be dropped from the model, and inferences about treatment response will be based on the main effect of treatment. If the treatment does indicate site-to-site variation in change in PDC, then pairwise comparisons between changes at the sites will be made using the Tukey honestly significant difference to control the type I error rate.

#### Aim 1a: To Assess Patient Engagement and Behaviors Related to the Use of InCharge Health

Counts and scores of the measures in Textboxes 1 and 2 will be graphed with box plots by month. Using the box plots from the last month, patients will be classified into 4 levels of app usability: *low* (<25% of the daily app usage), *medium-low* (25%-49% of the daily app usage), *medium-high* (50%-74% of the daily app usage) or *high* (75%-100% of the daily app usage) by initially using quartiles of the implementation measures, then examining the box plots of the measures and adjusting as needed to create 4 clinically meaningful groupings of app users. App uptake will be computed at the end of the study at each site.

#### Aim 1b: To Examine the Improvement in Clinical and Patient Outcomes Related to the Use of InCharge Health

The linear mixed model will also be employed to evaluate changes in laboratory biomarkers of hydroxyurea effect, quality of life, health literacy, self-efficacy, and satisfaction with the app (as listed in Textboxes 1 and 2) between baseline and 24 weeks. The models will include the treatment indicator and site as usual, a 4-level categorical predictor of use of the app (low, medium-low, medium-high, and high) and the interaction of app use with the treatment. A statistically significant interaction will be interpreted to mean that changes in an outcome in response to the treatment varied with use of the app. The Tukey HSD test will then be employed to identify the pairwise differences that contributed to the significant effect. If no variation with use of the app is found, then the interaction will be dropped from the model as described earlier. The models for laboratory biomarkers will also include time since starting hydroxyurea and sickle cell genotype as covariates. Models, including site-level characteristics of urban versus rural and academic versus community will also be created to determine if heterogeneity in the site characteristics impacts intervention efficacy. For dichotomous outcomes, such as PDC ≥80% versus <80% and hospitalization and emergency room visits during the study, generalized linear mixed models (GLMM) will be employed with a logit link to relate the outcome to the predictors [65]. The GLMM models will follow the same format as the linear mixed model above with fixed effects for intervention/app use and random effects for sites and subjects within sites.

#### Aim 2: Improve Provider Hydroxyurea Prescribing Behaviors

Given the limited number of providers expected to enroll in the study, many of the analyses are simplified and do not account for the across the site and across time complexities of the study design. As such, the results should be considered exploratory. Using baseline data, providers will be classified into 4 categories, according to the level of comfort and expertise in caring for patients with SCD (Multimedia Appendix 2). We will attempt to evaluate the implementation and effectiveness outcomes stratified by this provider categorization to better understand how expertise impacts the implementation and effectiveness of the *HU Toolbox* app.

#### Aim 2a: To Examine Clinic Characteristics and Provider Engagement and Behaviors Related to the Use of the HU Toolbox

**Figure 5:**
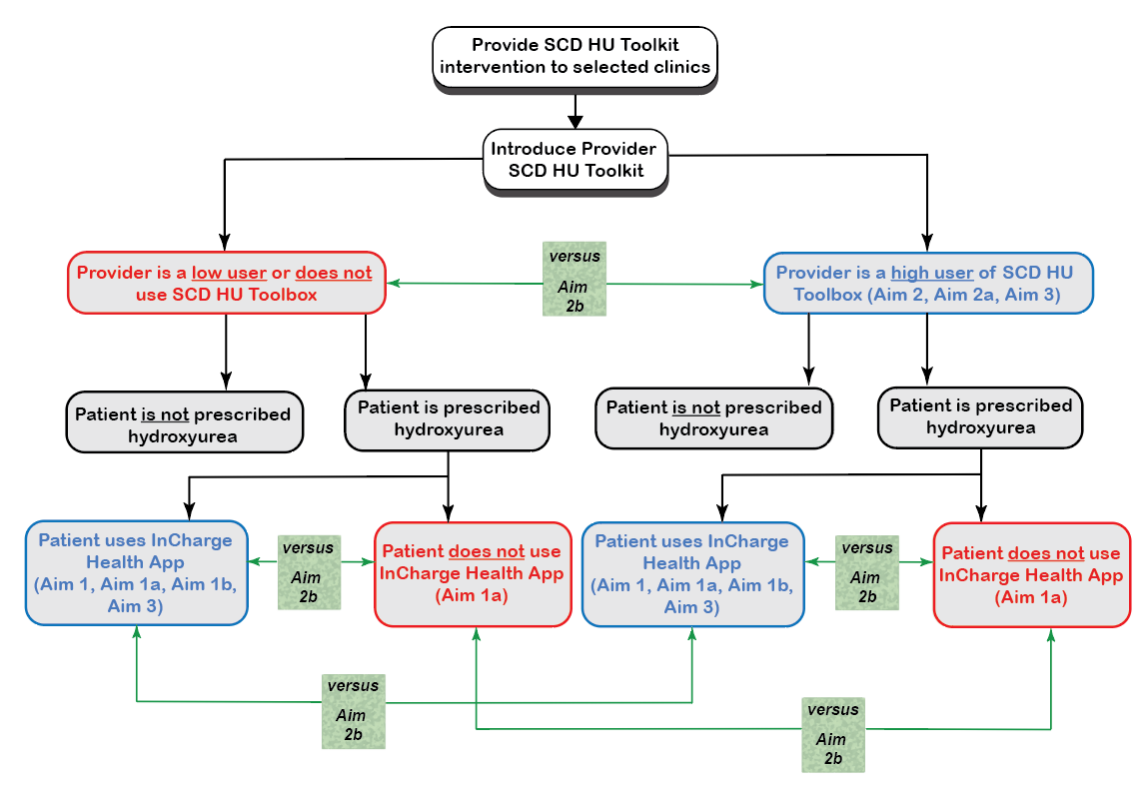
Study group comparisons according to each aim. The introduction and investigation of each intervention is performed sequentially. A total of 4 possible intervention combinations will be evaluated and compared: provider and patient use the intervention (patient and provider blue boxes), neither provider nor patient uses the intervention (patient and provider blue boxes), provider uses the intervention, but the patient does not (provider: blue box and patient: red box), and the patient uses the intervention, but the provider does not (provider red box and patient blue box). HU Toolbox: Hydroxyurea Toolbox; SCD: sickle cell disease.

Uptake of the *HU Toolbox* by providers after 9 months will be assessed using the implementation measures identified in Table 4 (under implementation). Box plots for each measure for all participants will be combined and stratified by expertise level. One-way analysis of variance or Kruskal-Wallis tests will examine differences in the uptake of the *HU Toolbox* across expertise levels (Figure 5). If the null hypothesis is rejected, Dunn’s test will be employed for multiple comparisons. If an experience level has fewer than 5 providers, it will be combined with the closest lower experience level. The results of these analyses will be used to identify clinically meaningful *low* and *high* toolbox app uptake groups for Aim 2b.

#### Aim 2b: To Assess the Combined Effects of the Patient and Provider Mobile Health Interventions on Hydroxyurea Adherence and Health Care Utilization

Figure 5 shows the sequential introduction and investigation of each intervention. A total of 4 possible intervention combinations will be evaluated and compared: provider and patient use the intervention, neither provider nor patient uses the intervention, the provider uses the intervention, but the patient does not, and the patient uses the intervention, but the provider does not. Comparisons within and across groups will be conducted. This analysis seeks to identify the impact of both the patient and provider interventions on hydroxyurea adherence and acute health care utilization (count of emergency department visits and hospitalizations per patient) at baseline and at 6 months. These outcomes will be treated as Poisson variables in GLMMs with log link functions. Predictors will include an indicator for time (baseline vs 6 months), a categorical predictor for the 4 levels of *InCharge Health* app uptake defined in Aim 1a, an indicator for low (less than one day per month use of the app in a 6-month period) versus high provider (one or more days per month use of the app in a 6-month period) toolkit app uptake, the interaction between patient and provider uptake, and a random effect to account for clustering of baseline and 9-month measures within providers. The Tukey HSD test will be employed for pairwise comparisons among predictor categories if statistically significant effects of patient characteristics, provider characteristics, or interactions are found. App uptake will be computed at the end of the study at each site.

#### Aim 3: Qualitatively Evaluate the Barriers and Facilitators of the Implementation of Mobile Health Interventions

Sufficient understanding of the contextual factors in the implementation of mHealth interventions is critical to ensuring future scale-up and translation of study findings [66]. As such, for Aim 3, we will build on the RE-AIM quantitative findings by using qualitative inquiry to identify common barriers and facilitators across the sites and to support the development of implementation strategies for use in future studies. Data will be collected and analyzed concurrently using a mixed methods approach, where qualitative data will be secondary to the quantitative assessment [67].

## Results

The study is currently enrolling participants (NCT 04080167). Recruitment is anticipated to be completed by mid-2021. The results are expected to be submitted for publication toward the end of the project in early 2022.

## Discussion

Hydroxyurea has proven its efficacy in treating patients with SCD, but its utilization in real-world settings is suboptimal. mHealth interventions have increasingly been used to foster greater adherence to medication and to facilitate the use of therapies by prescribers. In this study, we propose to overcome the barriers to hydroxyurea utilization by using a 2-level mHealth intervention: the *InCharge Health* app for patients and the *HU Toolbox* app for providers. We will examine the uptake of these 2 interventions using implementation science strategies. Although acknowledging the multilevel barriers to hydroxyurea utilization, our approach will address the main barriers affecting hydroxyurea adoption and use among patients with SCD and focus on improving prescribing practices among providers. This 2-level approach will allow us to demonstrate the clinical effect of mHealth interventions to improve adherence among patients (the main outcome of the study) and, at the same time, address and evaluate other barriers to optimal care among providers. Our findings will enhance the subsequent implementation of mHealth in diverse settings and populations, as the participating sites are substantially different in geographical settings (eg, urban, suburban, and rural) and population characteristics. The study will provide preliminary data on the integration of mHealth into clinical care, its clinical influence, and evaluate how well this strategy is accepted, adopted, and sustained in diverse clinical settings.

The Integration of mHealth into SCD Care to Increase Hydroxyurea Utilization study will be the first large-scale prospective trial to investigate mHealth interventions for SCD using an implementation science framework to improve hydroxyurea effectiveness and adaptation while incorporating implementation strategies to maximize the integration of the apps into clinical practice. If successful, this model may create a new paradigm in which mHealth interventions can be integrated into routine clinical practice in real-world settings. This approach focuses on the complexity of therapies for chronic diseases in which the lack of widespread adaptation is multifactorial and should account for multiple stakeholders.

This study has some limitations. PDC is one of the multiple measures of medication adherence; however, it allows us to pragmatically estimate adherence as it approximates optimal adherence when PDC is ≥80%. PDC is an accepted quality measure of adherence and the metric used by the Centers for Medicare and Medicaid Services as the process measure of adherence [68]. PDC best reflects the *real-world* setting as opposed to the use of electronic bottles or video-recorded daily dose ingestion (ie, directly observed adherence measure). The Integration of mHealth into SCD Care to Increase Hydroxyurea Utilization study does not include randomization of mHealth interventions, and this approach may be considered as the next step in our research.

Few prospective intervention studies to improve hydroxyurea adherence in SCD using mHealth have been undertaken [42]. In general, they have shown positive results in improving daily hydroxyurea utilization and other outcomes, such as health-related quality of life [69,70]. The Integration of mHealth into SCD Care to Increase Hydroxyurea Utilization study expands the existing studies because it (1) addresses all the important behavior determinants of suboptimal hydroxyurea utilization at both the patient and provider levels, (2) incorporates them into mHealth apps for both patients and providers, and (3) studies a large population of patients with SCD and their providers in different geographic and clinical settings. Finally, engagement with mHealth interventions will be of particular importance during the study, as sustained use of mHealth intervention may decrease over time. Our study plans to evaluate engagement with mHealth interventions by monitoring app usability (frequency of use and specific features used) in addition to performing qualitative analysis to better ascertain factors influencing engagement with mHealth.

In summary, the Integration of mHealth into SCD Care to Increase Hydroxyurea Utilization study is the first study investigating the efficacy and implementation of mHealth interventions on 2 levels to improve hydroxyurea utilization, namely the patient and the provider, in a large multicenter prospective study. Importantly, the development of both mHealth interventions was informed by the stakeholders involved (patients with SCD and their providers). If successful, this study will help define the role of mHealth in increasing hydroxyurea utilization at multiple levels and will allow for a large-scale implementation trial that will rigorously test which strategies are most effective in disseminating mHealth to more patients with SCD and their providers.

## Appendix

Multimedia Appendix 1 SPIRIT checklist.

Multimedia Appendix 2 Categorization of providers according to expertise level (classification developed by Dr Wally R Smith and Dr Richard Lottenberg).

Multimedia Appendix 3 List of sickle cell disease implementation consortium investigators.

## Abbreviations

ANOVA: analysis of variance
ED: emergency department
GLMM: generalized linear mixed models
HbF: fetal hemoglobin
HBM: health belief model
HU Toolbox: Hydroxyurea Toolbox
LDH: lactate dehydrogenase
LMM: linear mixed model
MARS: mobile app rating scale
MCV: mean corpuscular volume
mHealth: mobile health
MTD: maximum tolerated dose
NHLBI: National Heart, Lung, and Blood Institute
NIH: National Institutes of Health
PDC: proportion of daily coverage
PROMIS: Patient-Reported Outcomes Measurement Information System
RE-AIM: reach, effectiveness, adoption, implementation, and maintenance
SCD: sickle cell disease
SCDIC: sickle cell disease implementation consortium
SPIRIT: Standard Protocol Items for Clinical Trials
