# Supplementary figures and images for: Integration of Mobile Health Into Sickle Cell Disease Care to Increase Hydroxyurea Utilization: Protocol for an Efficacy and Implementation Study
Source: JMIR Res Protoc. 2020 Jul 14;9(7):e16319. doi: 10.2196/16319 (PMC7388044; doi:10.2196/16319)

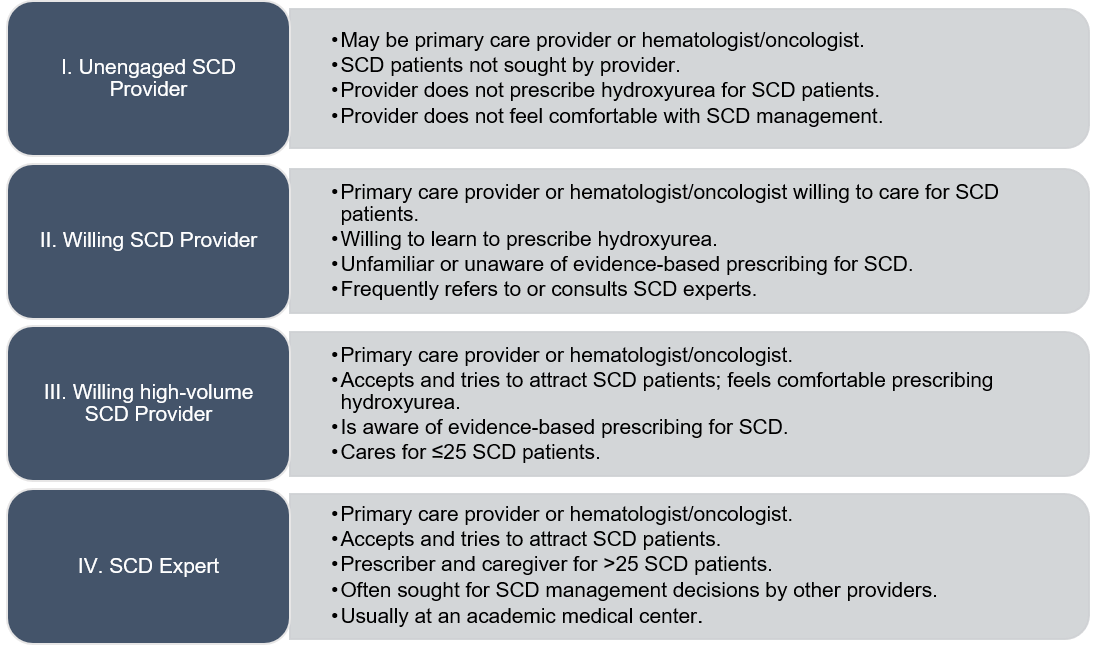

Supplement: Multimedia Appendix 2 [file resprot_v9i7e16319_app2.png]
