# Supplementary material for: Integration of Mobile Health Into Sickle Cell Disease Care to Increase Hydroxyurea Utilization: Protocol for an Efficacy and Implementation Study
Source: JMIR Res Protoc. 2020 Jul 14;9(7):e16319. doi: 10.2196/16319 (PMC7388044; doi:10.2196/16319)
Supplement: Multimedia Appendix 3 [file resprot_v9i7e16319_app3.docx]

St. Jude Children’s Research Hospital, Memphis, TN

Jane Hankins, MD, MS

Jason Hodges, PhD, MA

Yvonne Carroll, RN, JD

Lisa Klesges, PhD, MS

Hamda Khan, MA

Matthew Smeltzer, PhD, MS

Chinonyelum Nwosu, MPH

James Gurney, PhD

Jerlym Porter, PhD, MPH

Nicole Alberts, PhD

Jeffrey Frey, BS, MS, MBA, PhD

Reginald French

Michael DeBaun, MD, MPH

Ramin Homayouni, PhD

Tamanna Shamrin, PhD, MS

Guolian Kang, PhD

Jeremie Estepp, MD

Winfred Wang, MD

Artangela Henry, DNP

Curtis Owens, MD

Margaret Debon, PhD

Ray Osarogiagbon, MD

Nidhi Bhatt, MD

Justin Flowers

University of California, San Francisco, CA

Marsha Treadwell, PhD

Elliott Vichinsky, MD

Ted Wun, MD

Michael Potter, MD

Danielle Hessler, PhD

Ward Hagar, MD

Anne Marsh, MD

Lynne Neumayr, MD

University of South Carolina, Charleston, SC

Cathy Melvin, PhD

Julie Kanter, MD

Shannon Phillips, PhD, RN

Robert Adams, MD

Martina Mueller, PhD

DUKE University, Durham, NC

Nirmish Shah, MD

Paula Tanabe, PhD, MSN

Hayden Bosworth, PhD

George Jackson, PhD

Fred Johnson, MBA

Rachel Richesson, PhD

Janet Prvu-Bettger, ScD

Washington University, St. Louis, MO

Allison King, MD, PhD

Ana Baumann, PhD

CeCe Calhoun, PhD

AUGUSTA University, Augusta, GA

Richard Lottenberg, MD

Abdullah Kutlar, MD

Robert Gibson, PhD

Angie Snyder, PhD

Maria Fernandez, PhD

Ichan School of Medicine at Mount Sinai, New York, NY

Lynne D. Richardson, MD

Jeffrey Glassberg M.D. M.A.

Jena Simon, MS, APRN-BC

Nicholas G. Genes, MD, PhD

George T. Loo, DrPH

Jason S. Shapiro, MD, MA

Kimberly Souffront PhD, FNP-BC, RN

Cindy Clesca, MA

Elizabeth Linton, MPH

Gery Ryan PhD, MA

RTI INTERNATIONAL

Barbara L Kroner, PhD

Lucia Rojas-Smith, DrPH

Tabitha Hendershot, BA

Lisa DiMartino, PhD, MPH

Sara Jacobs, PhD

Whitney Battestilli, BA

Donald Brambilla, PhD

NHLBI, Bethesda, MA

Sharon M Smith, PhD

Harvey Luksenberg, MD

Marlene Peters-Lawrence, BSN, RN

Cheryl Boyce, PhD

Ellen Werner, PhD

LURIE CHILDRENS, Chicago, IL

Alexis Thompson, MD

Sherif Badawy, MD, MS, MBBCh

University of Chicago, Chicago, IL

Victor Gordeuk, MD

Melissa Gutierrez, MS

Jana Hirschtick, PhD

Lewis Hsu, MD, PhD

Jerry Krishnan, MD, PhD

Nadew Sebro, MD

Larissa Verda, MD, PhD

Abe Wandersman, PhD

Michael Berbaum, PhD

Kishore Bobba, MD

Joe Colla, MD

Kim Erwin, MDes

Andrea Lamont, PhD

Molly Martin, MD. MAPP

Sarah Norell, MDes, MFA

Ananta Pandit, MD

Kay Saving, MD

Robin Shannon, DNP, RN

Robert Winn, MD

Leslie Zun, MD

Taif Hassan, MD

Patricia Lasley, MPH

Kristin Monnard, MPH

Judith Nocek, PhD

Pamela Roesch, MPH
